# Accuracy Analysis of Deep Learning Methods in Breast Cancer Classification: A Structured Review

**DOI:** 10.3390/diagnostics13040683

**Published:** 2023-02-11

**Authors:** Marina Yusoff, Toto Haryanto, Heru Suhartanto, Wan Azani Mustafa, Jasni Mohamad Zain, Kusmardi Kusmardi

**Affiliations:** 1 Institute for Big Data Analytics and Artificial Intelligence (IBDAAI), Kompleks Al-Khawarizmi, Universiti Teknologi MARA (UiTM), Shah Alam 40450, Selangor, Malaysia; 2 College of Computing, Informatic and Media, Kompleks Al-Khawarizmi, Universiti Teknologi MARA (UiTM), Shah Alam 40450, Selangor, Malaysia; 3 Department of Computer Science, IPB University, Bogor 16680, Indonesia; 4 Faculty of Computer Science, Universitas Indonesia, Depok 16424, Indonesia; 5 Faculty of Electrical Engineering Technology, Universiti Malaysia Perlis, UniCITI Alam Campus, Sungai Chuchuh, Padang Besar 02100, Perlis, Malaysia; 6 Department of Anatomical Pathology, Faculty of Medicine, Universitas Indonesia/Cipto Mangunkusumo Hospital, Jakarta 10430, Indonesia; 7 Human Cancer Research Cluster, Indonesia Medical Education and Research Institute, Universitas Indonesia, Jakarta 10430, Indonesia

**Keywords:** breast cancer, classification, histopathological image, review

## Abstract

Breast cancer is diagnosed using histopathological imaging. This task is extremely time-consuming due to high image complexity and volume. However, it is important to facilitate the early detection of breast cancer for medical intervention. Deep learning (DL) has become popular in medical imaging solutions and has demonstrated various levels of performance in diagnosing cancerous images. Nonetheless, achieving high precision while minimizing overfitting remains a significant challenge for classification solutions. The handling of imbalanced data and incorrect labeling is a further concern. Additional methods, such as pre-processing, ensemble, and normalization techniques, have been established to enhance image characteristics. These methods could influence classification solutions and be used to overcome overfitting and data balancing issues. Hence, developing a more sophisticated DL variant could improve classification accuracy while reducing overfitting. Technological advancements in DL have fueled automated breast cancer diagnosis growth in recent years. This paper reviewed studies on the capability of DL to classify histopathological breast cancer images, as the objective of this study was to systematically review and analyze current research on the classification of histopathological images. Additionally, literature from the Scopus and Web of Science (WOS) indexes was reviewed. This study assessed recent approaches for histopathological breast cancer image classification in DL applications for papers published up until November 2022. The findings of this study suggest that DL methods, especially convolution neural networks and their hybrids, are the most cutting-edge approaches currently in use. To find a new technique, it is necessary first to survey the landscape of existing DL approaches and their hybrid methods to conduct comparisons and case studies.

## 1. Introduction

Cancer, which occurs when cells in the body grow unnaturally, is one of the main causes of human death. It has become a huge problem that threatens the safety and well-being of people all over the world. Breast cancer is one of the most widely known types of disease affecting women. These days, breast cancer is the deadliest cancer that can strike women, making it the leading cause of death overall. Due to its high prevalence and broad dissemination, breast cancer is dangerous and mostly depends on pathological diagnosis [1]. Accurate breast cancer diagnoses can improve cancer survival rates. Imaging modalities are being developed to promptly detect this disease. Cancer diagnosis relies on medical images, which are used for screening, diagnostic, and adjunctive evaluation to give field experts more confidence in their initial decision. Mammography, histopathology, MRI, ultrasonography, thermography, and PET/CT are imaging modalities. Each modality has its own advantages and disadvantages [2]. For instance, a mammogram is suitable for early detection. However, it is expensive and has low specificity.

Histopathological images serve as a valuable guide for the pathological analysis of this disease, as they can be used to describe tumor characteristics in all subgroups of interval cancers. A precise prognosis is dependent on the accurate classification of these images. It has been shown that the histopathological tumor characteristics of missed interval cancers are worse than those of true interval cancers in terms of prognosis [3]. For this reason, the number of interval cancers in a screening program can be reduced by improving a radiologist’s perception and interpretation by establishing a systematic collection of features and implementing structured reviews. Much research on breast cancer has been conducted to identify benign and malignant categories, with histopathological imaging often used in diagnosis. However, the high complexity of histopathological images and their associated heavy workload makes this work extremely time-consuming [3,4,5]. Additionally, diagnosis is tough, routine, and repetitive, as the clinical diagnosis of breast cancer is a complex problem. Additionally, the subjectivity of each pathologist may influence results, further complicating matters.

Histopathological breast cancer image recognition is a difficult task [6,7]. Traditional manual feature extraction and image classification usually necessitate in-depth knowledge, and more professional researchers are needed to extract and calculate high-quality image features. This operation typically takes a long time and produces poor classification results. It is critical in this field to develop an accurate, automated method for analyzing histopathological images because breast cancer diagnosis heavily relies on histopathological image classification [8]. Furthermore, previous research has shown that histopathological images have important clinical implications in the early stage of breast cancer screening [9,10]. The accurate classification of histopathological images could be used to develop automated and precise breast cancer classification systems for early detection [9]. Therefore, the proper screening of histopathological images is critical for the earlier detection and diagnosis of breast cancer.

Much work on histopathological image classification has been reported. Decades ago, traditional machine learning (ML) approaches, such as artificial neural networks (ANNs), support vector machines (SVMs), and decision trees, began to be used in breast cancer image classification. Deep learning (DL) has been introduced to overcome issues of accuracy in ML models. DL has snowballed and been successfully applied to many applications [11]. DL allows for massive amounts of data learning [12,13,14]. More importantly, DL has improved sound and image recognition [15,16,17]. DL comprises several methods, such as convolution neural networks (CNNs), recurrent neural networks (RNNs), long short-term memory (LSTM), gated recurrent unit (GRU), and bidirectional LSTM [15,16,17]. The CNN is a popular DL method that directly learns from input without feature extraction. The CNN is famous for being used to improve regularized MLP networks. CNN layers optimize parameters for meaningful output and reduce model complexity. Visual geometry group (VGG), AlexNet, Xception, Inception, ResNet, and other CNN variants can be used in different application domains depending on their learning capabilities [18,19]. Another popular DL method is the RNN. The CNN and RNN architectures differ. RNN works with feedback results [15,20]. Time series data mostly require an RNN where feedback loops provide memory for optimal performance in context-sensitive data [21], such as sentence completion.

As a result, this study examined recent technological advances in histopathological breast cancer image classification using DL methods. Consequently, this research considered DL methods to evaluate the performance of current classification solutions. Researchers have proposed DL techniques to determine the best method for classifying histopathological breast cancer images, and these techniques were reviewed in this paper. The findings of this study are expected to assist researcher in choosing a suitable DL method. Correct diagnoses are required for complete care in a limited amount of time in cases of breast cancer, as the classification of benign and malignant cancers can save lives. DL performance depends on an image’s type, size, and features. Many previous studies embedded augmentation operations or strategies into DL methods to improve classification accuracy for histopathological images, with several methods employed to enhance image features.

While DL methods have shown promise, there is a need for a recent review of their efficacy in analyzing histopathological breast cancer images solutions. This work investigated DL and integration with other feature extraction, normalization, and optimization methods in histopathological image classification. This study employed a research ideology review to determine the best way to classify breast cancer based on its histopathology. The review considers what is known now and what methodological contributions have been made to classification solutions for specific problems. The rest of the paper is organized as follows. Section 2 describes the review methods. Section 3 shows the results and findings. Section 4 concludes the discussion.

## 2. Review Method

The use of contemporary technology in medical data analysis has evolved along with technology, particularly in image processing, classification, and segmentation, as well as in cancer research. The most well-liked machine learning method for medical image diagnosis, DL, is being used by an increasing number of researchers. The medical community agrees that the future of DL in disease prediction is promising [22]. Additionally, scientists have applied various classification techniques to cancer image data to categorize breast cancer. Convolutional neural networks (CNNs) are widely used in image classification [23,24]. In addition, many researchers have tried to apply pre-processing, feature extraction, ensemble learning, and classifier techniques to automate the detection of breast cancer cells.

High-performance evaluation values in terms of accuracy, specificity, sensitivity, precision, and F-measure can be obtained using several methods, and DL methods can be used to compensate for traditional methods of breast cancer diagnosis, allowing for the early detection of the disease. This paper provides a comprehensive review based on advanced searching related to the classification of histopathological breast cancer images using DL and its hybrids. Advanced evaluation is one of the most critical discussions at the moment. Thus, a systematic flow method was used in this work. A protocol or plan with clearly stated criteria before a review is referred to as a structured review [25], which is a method for strategically identifying patterns, trends, and critical evaluations of the literature on research subjects [26].

**Figure 1:**
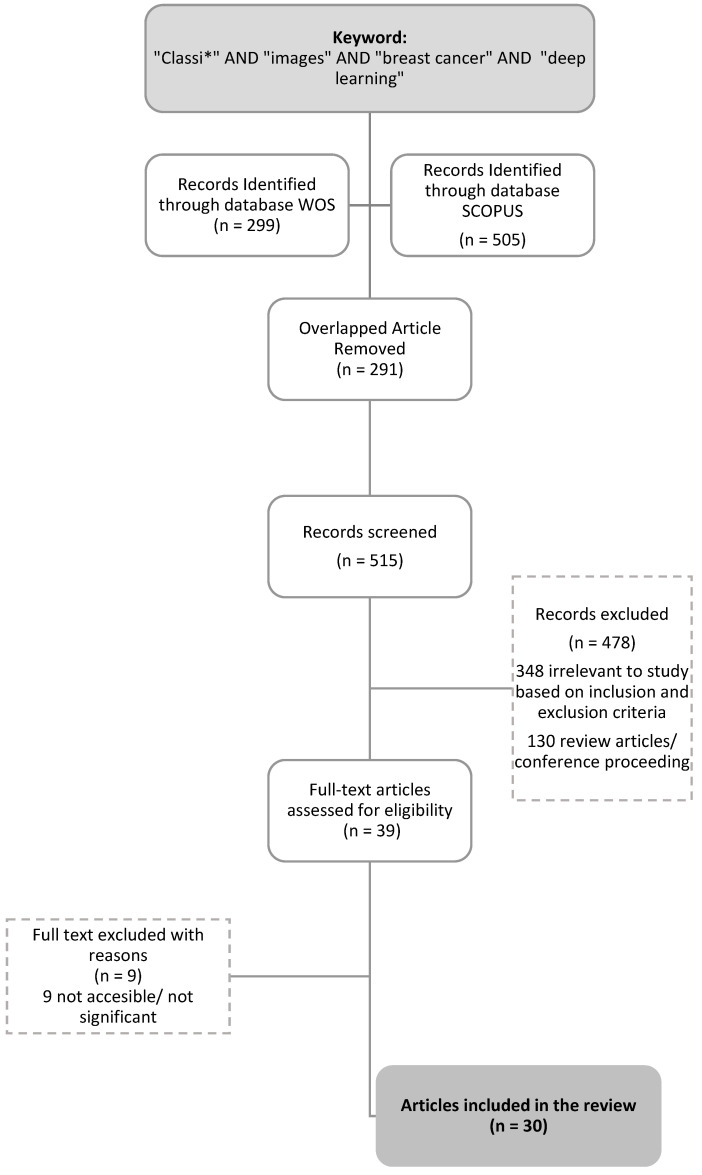
The PRISMA flow diagram of the entire procedure for selecting reviewed articles.

Following the analysis and integration of the results, further readings in the literature were utilized to develop future research directions on the applications of DL to histopathological classification. The review technique comprised four steps for choosing numerous relevant papers for this study. This study used the Preferred Reporting Items for Systematic Reviews and Meta-Analyses (PRISMA) methodology [27]; it is a framework created to illustrate the flow of information during the various stages of a systematic review, as shown in Figure 1. The first step in writing this comprehensive literature review was identifying research items that may be relevant to the research question. The total number of searched papers was then screened. Finally, the eligibility of each paper based on its abstract was evaluated. Overall, the scientific literature was reviewed and summarized to identify, select, and evaluate breast cancer classification techniques. Subsequently, additional research directions to address the raised concerns were recommended. In this study, the best practice method was used to conduct the comprehensive literature review, and the publication rules provided essential information to help the researchers evaluate the accuracy of the review. Furthermore, an investigation for the systematic analysis of the various studies considered within this review. The WOS and Scopus databases were used to examine the studied methodologies.

### 2.1. Preliminary Identification

Articles in the literature were identified to select studies on the utilization of DL in histopathological classification. Keywords such as “classi*” AND “image*” AND “breast cancer” AND “deep learning” were applied. The year was restricted to 2022 to consider all related recent studies. The literature search was conducted by using the Scopus and WOS databases. The initial search yielded 505 articles from Scopus and 299 articles from the WOS, as demonstrated in Table 1.

### 2.2. Screening

Screening is used to examine relevant research items for content that matches predefined research question(s). Here, machine learning-classified cervical cancer cells were employed to select research items in the screening phase. This step removed duplicate papers from the searched list. The first screening eliminated 291 publications, and the second step comprised the examination of 515 papers based on this study’s exclusion and inclusion criteria (see Table 2). The selection criteria for the search were based on those of Alias et al. [28]. Research papers were the first criterion because they provide practical advice, and they include reviews, meta-synthesis, meta-analyses, books, book series, chapters, and conference proceedings not included in the most recent studies. Publications written in English released in 2022 were analyzed and considered for coverage. Note that 478 articles were removed because of their premature results and because they did not discuss the DL method in histopathological classification. Some articles were also incomplete, and some of the full articles were not accessible, had broken links, and overlapped.

### 2.3. Eligibility

After all inclusion and exclusion criteria were met, the final review sample was generated. Based on the research objectives, the inclusion criteria included articles that helped us find recent solutions for challenges in histopathological breast cancer imaging, DL methodology, and types of classification analysis. Consequently, 9 publications were excluded since their titles and abstracts were not significantly related to the study’s purpose based on empirical data. Finally, 30 papers were made available for evaluation (see Figure 1).

**Figure 2:**
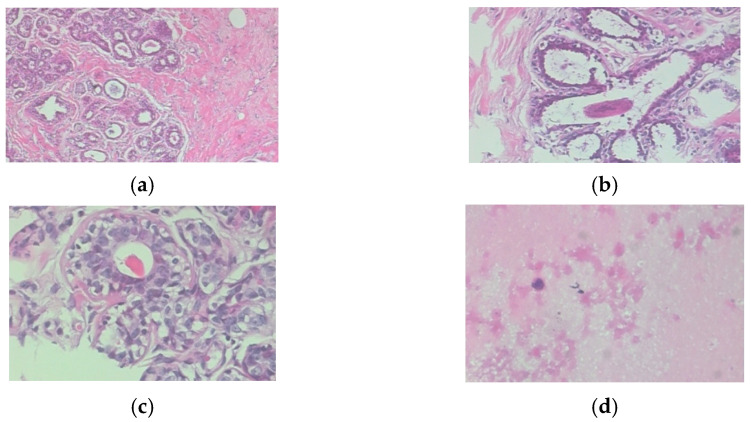
(**a**) Histopathological breast cancer images of the BreakHis dataset: (**a**) 40×; (**b**) 100×; (**c**) 200×; (**d**) 400×.

A further inclusion criterion was that the studies had to be in the field of computer science and engineering. This helped narrow this review to DL theories and methods in histopathological breast cancer classification. Furthermore, tools and predictive factors aided the extraction, comparison, and synthesis processes. The exclusion criteria excluded articles that focused on different contexts (such as brain cancer, traditional machine learning, and healthcare) and studies that were not specifically about histopathological breast cancer images. Figure 2 shows examples of four magnification classes of histopathological images from the BreakHis dataset. Then, the authors of this study generated themes based on evidence from this analysis. Analyses, points of view, questions, and other data interpretation ideas were recorded in a log. Finally, the authors looked for theme design inconsistencies in the results; they discussed any conceptual differences, and themes were tweaked to ensure consistency. To validate the issues and ensure subtheme clarity, importance, and appropriateness during the expert review, Dr. Kusmardi from Cipto Mangunkusumo Hospital, Jakarta, and the Faculty of Medicine, Universitas Indonesia, Indonesia, was chosen as a pathology expert.

## 3. Results and Findings

As the prevalence of breast cancer has grown, image-based histopathological analysis has become widely used in pathological research and disease diagnosis. However, pathologist errors in cell identification have been identified as a significant problem. As a result, a comparative evaluation of the proposed model was performed to demonstrate the utility of feature selection and class imbalance. In addition, how well the classifier performed in terms of accuracy, sensitivity, precision, F-measure, and specificity was assessed. This study evaluated the use of a cell classification algorithm associated with imaging in breast cancer screening to improve the effectiveness and accuracy of the early clinical diagnosis of breast cancer, and it was found that researchers have developed several methods to fix the problems of the proposed classification methods. One popular technique for categorizing cancer cells is the use of CNNs. Note that 30 articles were ultimately selected for inclusion and analysis based on extensive searching.

Classification of Histopathological Images on Deep Learning Approach

Many researchers have contributed to the field of histopathological image classification, and a summary of methods and outcomes facilitated comparisons between studies. Table 3 summarizes recent DL research on categorizing histopathological breast cancer images.

**Figure 3:**
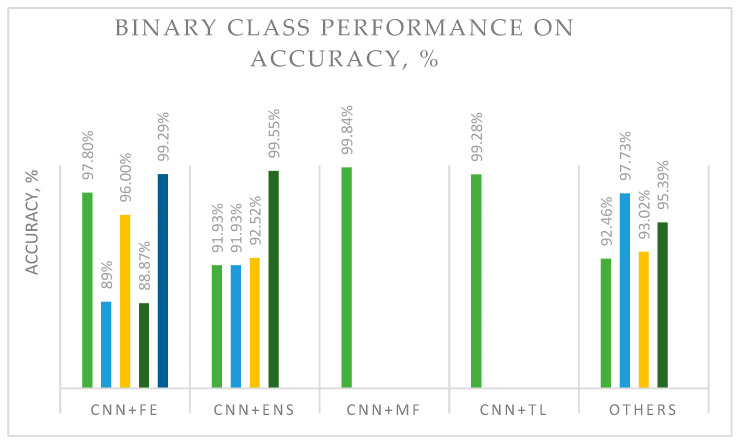
Binary classification accuracy of the popular and best classification approaches.

One plausible explanation for these findings is that the histopathological diagnosis of breast cancer classification has been researched several times. CNNs currently comprise one of the best approaches for the classification process. Previous researchers have studied several techniques related to CNNs, such as CNNs with ensembles, CNNs with feature extraction, CNNs with model fusion, and CNNs with classifiers. For example, Li et al. [1] introduced a novel model fusion framework based on knowledge transfer (MF-OMKT) for binary histopathological breast cancer image classification. It demonstrated better performance than that reported in other studies, with an accuracy of 99.84%. Their findings showed an improvement of 0.55% compared with a CNN with feature extraction and normalization. On the other hand, Karthik et al. [41] calculated the same accuracy of 99.55% with two ensemble learning strategies, namely, channel and spatial attention, integrated with custom deep architectures. The ensemble model reduced the margin of error and improved the overall classification performance. This kind of approach is usually called ensemble learning and involves fusing multiple learning algorithms. Ensemble learning could produce a robust and reliable model with good generalization performance. However, the results were still low in some cases, as seen in Figure 3.

Another improved model used CNN with pre-processing, showing accuracies of 0.44% for 40× and 400× magnifications, respectively, of the BreakHis dataset. These results were nearly identical to those of a previous study by Li et al. [1] who utilized CNN and filtering; LightXception achieved an accuracy of 97.42%, a recall of 97.42%, and a precision of 97.42% in that study. Another study by Yang and Guan [9,10] classified pathological medical images of breast cancer using the BreakHis image dataset and an improved network DenseNet201-MSD model; the new DL model classified pathological images of the BreakHis dataset with accuracies of 99.4%, 98.8%, 98.2%, and 99.4% at four magnifications. These results were consistent with those of [48], which used CNN with transfer learning and integrated Manta Ray Foraging Optimization (MRFO) as a metaheuristic optimization method to increase the adaptability of image features. As a nature-inspired algorithm, Manta Ray Foraging improved classification performance, but it requires more testing on several parameters. Additionally, Burçak and Uuz [52] concluded that CNN models are robust feature selection strategies in four categories of histopathological images. Their findings contradicted the findings of Rashmi et al. [36], who proposed a classification method based on a CNN and a color channel with an attention module (CWA-Net).

Amin et al. [22] proposed a hybrid semantic model that employed pre-trained Xception and deeplabv3+ models to classify microscopic cancer images into malignant and benign classes, with 95% and 99% accuracy for benign and malignant, respectively. Compared with previously published methodologies, the proposed framework demonstrated exceptional performance. Numerous scholarly articles have studied the classification and analysis of breast cancer using histopathological images. In addition, histopathological images of breast cancer patients are increasingly classified using deep CNNs. Most of the research solutions dealt with histopathological breast cancer modalities. The BreakHis dataset has mainly been popular in the binary classification of malignant and benign cancers. Different methodological approaches have demonstrated a variety of accuracy levels for the studied datasets. Much more research is expected to consider sub-classes of datasets, such as regarding a diversity of magnification to improve the performance of models, as seen in Table 3.

**Figure 4:**
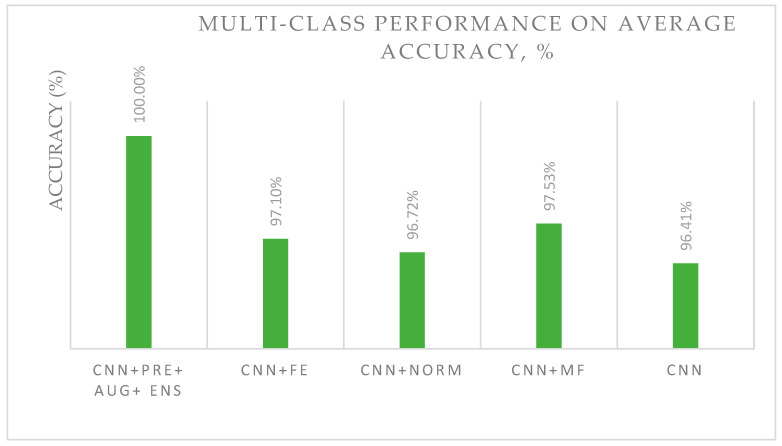
Multi-class classification accuracy of the popular and best classification approaches.

The authors of this study established two categories of classification approaches, namely, binary and multi-class DL solutions. Figure 3 and Figure 4 illustrate these two approaches, with a focus on the best performance and popular hybrid solutions. Hybrid DL models were found to demonstrate better accuracy in pathological image classification. For binary classification, five different methods were used in recent studies: a CNN with feature extraction (CNN + FE), a CNN with ensemble (CNN + ENS), a CNN with model fusion (CNN + MF), a CNN with transfer learning (CNN + TL), and others. Binary classification is based on popular BreakHis image data. It can be seen from Figure 3 that the CNN + FE and CNN + ENS models have been the most popular approaches in the binary classification of breast cancer based on histopathological images. Additionally, the classification method based on CNN with model fusion demonstrated a higher accuracy level (more than 99.84%) than other studied approaches, as illustrated in Figure 3. In this case, fusion adaptation showed significant influence on feature extraction. Other approaches showed accuracies ranging from 89% to 99.55%. For instance, the recent DL with ensemble approach demonstrated an accuracy of 91% to 92%. Therefore, more research is required to improve model classification accuracy, although the CNN + MF model has been shown to be the best of the currently described methods. In addition, statistical analysis methods such as the t-test must be performed to confirm the significance of the studied methods.

For multi-class classification, studies have used a CNN with pre-processing, augmentation, and ensemble (CNN + PRE + AUG + ENS); a CNN with feature extraction (CNN + FE); a CNN with model fusion (CNN + MF); a CNN with normalization (CNN-Norm); and a CNN alone. Figure 3 demonstrates the performance of DL variants for multi-class histopathological image classification. The CNN + PRE + AUG + ENS model outperformed other methods, with an accuracy of 100%.

## 4. Discussion

DL technological advancements are propelling the growth of automated breast cancer diagnosis. A breast cancer diagnosis can be performed with various image modalities, such as histopathological images. One of the most significant challenges in DL has long-been the accurate and automatic classification of pathological medical images. Additionally, the use of deeper layers in neural networks enables higher abstraction levels and more precise data analysis. Therefore, neural networks have become increasingly popular in evaluate the performance of classification approaches in recent years. Researchers have proposed many CNN variants to determine the best method for classifying histopathological images, as discussed in this paper. The findings of this study are intended to help identify the best performing CNN methods.

Several established methods can be used to detect and classify benign and malignant cancers based on deep feature characteristics. Ensemble learning and embedded fusion models have shown better performance than other integration methods. Furthermore, a CNN with model fusion is a powerful tool for precise feature extraction and histopathological image classification. The suggested idea of adapting an online mutual knowledge transfer strategy as a fusion strategy embedded in CNNs could be promising for other types of breast cancer detection.

Different levels of accuracy were demonstrated by several hybrid CNN methods. Some showed an excellent accuracy of more than 97%, and others showed an accuracy of below 97%. The combination of a CNN with FE and ENS has shown different levels of accuracy for the same dataset depending on the data variance, feature selection, and methodological approach used in binary and multi-class classification. It is evident that the fusion strategy has a high viability in binary classification solutions but a low viability for multi-class classification solutions. Another promising strategy with strong performance is the combination of the pre-processing, augmentation, ensemble, and CNN methods.

The progression of DL has resulted In the production of promising solutions for the binary and multi-class classification of breast cancer images, with a primary focus on histopathology. It is hoped that more health information on areas such as the brain, eyes, chest, heart, abdomen, musculoskeletal system, and other human body regions will be incorporated into DL models. The findings presented in Figure 3 and Figure 4 and Table 3 can serve as a foundation for developing DL models. Incorporating pre-processing, feature extraction, and augmentation methods into models is one way to improve their performance. In addition, the use of a fusion strategy is likely to produce favorable outcomes for binary classification and could be improved to suit multi-class image data.

## 5. Conclusions

A correct diagnosis is necessary for the comprehensive treatment of breast cancer in a short time. Accordingly, lives can be saved with the accurate classification of benign and malignant cancers. DL performance depends on the input images’ type, size, and characteristics. Many previous studies embedded augmentation operations or strategies into DL methods, especially CNNs, to improve classification accuracy for histopathological images. Similarly, several methods have been proposed to enhance image features. However, a review of DL models and their performance for histopathological breast cancer images is lacking for both binary and multi-class classification solutions. Therefore, this work investigated DL and its integration with other feature extraction and normalization methods in histopathological image classification. This review paper is intended to aid the creation of better breast cancer classification designs and methodologies to assist in the identification process of this cancer. Furthermore, the proposed CNN hybrid architecture simplifies the detection and classification of cancer cells in histopathological images, potentially leading to the earlier detection of breast cancer and an increase in women’s survival rates. More research should be conducted on methods, beginning with studies of pre-processing, feature extraction, and classification using various breast cancer images. Furthermore, a new strategy for improving classification performance in histopathological images should be imposed on hybridization with computational optimization algorithms such as cuckoo search, the firefly algorithm, and particle swarm optimization to find local and global image features that lead to better classification performance.

## Figures and Tables

**Table 1:** Search strings from the Scopus and WOS databases.

Search strings from the Scopus database	TITLE-ABS-KEY “classi*” AND “images” AND “breast cancer” AND “deep learning” (LIMIT-TO PUBYEAR, 2022)	Results = 503 Articles
Search strings from the WOS (Web of Science) database	TS = “classi*” AND “images” AND “breast cancer” AND “deep learning” AB = “classi*” AND “images” AND “breast cancer” AND “deep learning” Refined by: DOCUMENT TYPES: (ARTICLE) AND PUBLICATION YEAR: 2022	Results = 299 Articles

**Table 2:** Second-stage examination criteria.

Criterion	Inclusion	Exclusion
Language	English	Non-English
Published Year	2022	<2022
Source type	Journal (only research articles)	Conference proceeding
Document Type	Article	Letter, review, conference, and note
Research Area	Computer Science and Engineering	Besides Computer Science and Engineering

**Table 3:** Summary of recent deep learning research.

Reference	Dataset	Class	Methodology	Results	Advantages/Shortcomings
[5]	BreakHis	Multi-class	Data augmentation and handcrafted feature extraction (FE) techniques (Hu moment, Haralick textures, color histograms, and deep neural networks (DNNs)). Hybrid DL method: CNN + feature extraction.	Accuracy: 40×: 97.87%; 100×: 97.60%; 200×: 96.10%; 400×: 96.84%. Average accuracy: 97.10%	The proposed handcrafted feature extraction and DNN showed the best performance.
[29]			Dual residual block with a multi-scale dual residual recurrent network (MTRRE-Net74). DL Method: RNN.	Accuracy: 40×: 97.12%; 100×: 95.2%; 200×: 96.8%; 400×: 97.81%.	Better accuracy at four magnification levels than previously described models.
[30]			NetDense residual dual-shuffle attention network (DRDA-Net) inspired by the bottleneck unit of the ShuffleNet architecture. DL Method: CNN.	Accuracy: 40×: 95.72%; 100×: 94.41%; 200×: 97.43%; 400×: 98.1%/	The proposed model showed acceptable accuracy. Densely connected blocks addressed the overfitting and vanishing gradient problems.
[3]			DenseNet201 and VGG16 architecture models as an ensemble model used to extract global features. DEEP Pachi extracts spatial information on the region of interest. Hybrid DL method: CNN + pre-processing + augmentation + ensemble.	BreakHis Malignant Accuracy: 99%. Benign Accuracy: 100%.	A significant result.
[31]	Wide-scale data		DeepML framework to achieve multi-class classification. DL Method: CNN.	Average accuracy: 98% (90–10% train–test split) and 89% (80–20% train–test).	Acceptable accuracy.
[32]	BreakHis	Binary	DL are DenseNet_201, MobileNet_V2, and Inception_V3. Ensemble or boosting methods are AdaBoost (ADB), Gradient Boosting Machine (GBM), LightGBM (LGBM), and XGBoost (XGB) with Decision Tree (DT) as a base learner. Hybrid DL method: CNN + ensemble.	XGB + Inception_V3 showed the best accuracy of 92.52%.	Feature extraction and boosting ensembles were shown to be a good combination for image classification.
[10]			Pre-processed by multiple scaling decompositions to prevent overfitting due to the large dimension. It improved the DenseNet-201-MSD network model. DL Method: CNN + preprocessing.	Accuracy: 40×: 99.4%; 100×: 98.8%; 200×: 98.2; 400×: 99.4%.	The mode demonstrated very good results and can be used with other image data.
[33]			LightXception is based on cutting off layers at the bottom of the Xception network, which reduces the number of convolution filter channels. LightXception only has about 35% of the parameters of the original. Hybrid DL method: CNN + filter.	At 100× magnification: Xception Accuracy: 97.31%; Xception Recall: 98.67; Xception Precision: 99.26%; LightXception Accuracy: 97.42%; LightXception Recall: 97.42%; LightXception Precision: 97.42%.	Acceptable classification solution; the model needs improvement.
[34]			Support vector machine (SVM), CNN, and CNN with transfer learning (TL). Hybrid DL method: CNN + TL.	Accuracy: SVM: 92%; CNN: 94%; CNN + TL: 97%.	Improved results for CNN + transfer learning compared with a single method.
[35]			DenseNet201 with the SVM RBF classifier. Hybrid DL method: CNN + classifier.	At 200× magnification: Accuracy of 95.39% and precision of 95.43%.	Acceptable accuracy could have been due to the use of traditional machine learning as a classifier.
[23]	BreakHis	Binary	The attention model features multi-scale channel recalibration and msSE-ResNet convolutional neural network (msSE-ResNet34). Hybrid DL method: CNN + FE.	Accuracy: 88.87%.	Accuracy was low, although the approach was embedded with FE.
[36]			CNN and color channel with attention module (CWA-Net). Hybrid DL method: CNN + FE.	At 400× magnification: Private dataset accuracy: 95%; BreakHis dataset accuracy: 96%	Acceptable accuracy.
[37]			DenseNet as a backbone model and transfer learning (DenTnet). Hybrid DL method: CNN + TL.	Accuracy: 99.28%.	Good generalization ability and computational speed.
[38]			“Deep-Hist” with pre-trained and Stochastic Gradient Descent (SGD). Type: CNN + optimization.	Accuracy: 92.46%.	The proposed model required accuracy improvements.
[39]			Pre-trained Xception model with VGG16 and enhanced with logistic regression. Uses real-time data augmentation (AUG). Hybrid DL method: CNN + TL + AUG.	Xception + VGG16: Precision of 78.67% and recall of 0.76. F-score of 0.75, and AUC of 0.86. Xception + VGG16 + logistic regression: precision of 82.45%, recall of 0.82. F-score of 0.82, and AUC of 0.90.	The proposed model outperformed a conventional CNN. Augmentation could reduce the problem of overfitting.
[22]	Breaches’	Binary	Xception and deeplabv3+. DL method: CNN.	Binary accuracy: 95%. Malignant accuracy: 99%.	The proposed framework demonstrated remarkable performance on a malignant dataset.
[40]			Inception-ResNet-v2 and Categorical Boosting (CatBoost), Extreme Gradient Boosting (XGBoost), and Light Gradient Boosting Machine (LightGBM). DL method: CNN.	Accuracy: 40×: 96.82%; 100×: 95.84%; 200×: 97.01%; 400×: 96.15%; Average: 96.46%.	The proposed Inception-ResNet-v2 and Categorical Boosting (CatBoost) models outperformed other methods.
[41]			Two custom deep architectures, CSAResnet and DAMCNN, integrated with channel and spatial attention. Type: CNN + ensemble.	Accuracy: 99.55%.	The proposed model demonstrated outstanding accuracy, showing that the employed ensemble method could be successfully used on the studied type of images.
[42]	Invasive breast carcinoma		CNN with Resnet50 and Xception. Type: CNN.	Xception was better than Restnet50, with an accuracy of 88% and a sensitivity of 95%.	Model performance was still low for Xception. The model could be improved by embedding ensemble and classifier programs.
[43]	BACH, UC, and BreakHis		Deconv-Transformer (DecT) with color jitter data augmentation. Hybrid DL method: CNN + AUG.	BreakHis dataset accuracy: 93.02%. BACH dataset accuracy: 79.06%. UC dataset accuracy: 81.36%.	Performance was low even though augmentation was added. A hyperparameter tuning and pre-processing method would improve performance.
[44]			Embeds attention mechanism and high-order statistical representation into a residual convolutional network (attention high-order deep network (AHoNet). Adds non-dimensionality reduction and local cross-channel interaction to achieve local salient deep features with normalization (NORM). Hybrid DL method: CNN + FE + NORM.	BreakHis dataset accuracy: 99.29%. BACH dataset accuracy: 85%.	Performance was more competitive than previously described models. A very good model to be tested with other types of image data.
[45]	BreakHis and FNAC	Binary	Twenty-eight hybrid architectures combining seven recent deep learning techniques for feature extraction (DenseNet 201, Inception V3, Inception ResNet V2, MobileNet V2, ResNet 50, VGG16, and VGG19) and four classifiers (MLP, SVM, DT, and KNN) for binary classification. Hybrid DL method: CNN + classifier.	DenseNet 201 (MDEN) with MLP showed the best performance. FNAC accuracy: 99.29%. BreakHis accuracy: 40×: 92.61%; 100×: 92%; 200×: 93.93%; 400×: 91.73%.	The proposed model requires accuracy improvements.
[46]	Public image database		Ensemble (ENS) for color adjustment methods with VGG-19 architectures. Hybrid DL method: CNN + ENS.	Accuracy: 91.93%. AUC: 97.72%.	The proposed model with color adjustment was shown to be an acceptable classification solution.
[47]	PCam Kaggle		Hybrid deep learning (CNN-GRU). Hybrid DL method: CNN and GRU.	Accuracy: 86.21%. Precision: 85.50%. Sensitivity: 85.60%. Specificity: 84.71%. F1-score: 88%. AUC: 0.89.	The proposed model showed low accuracy. GRU seemingly could not improve the method’s performance.
[48]	Histopathological data and ultrasound data		Automatic framework for reliable breast cancer classification CNN and TL. Embeds Manta Ray Foraging Optimization (MRFO) as metaheuristic optimization (OPT) to improve the framework’s adaptability. Hybrid DL method: CNN + TL + OPT.	Histopathological data accuracy: 97.73%. Ultrasound data accuracy: 99.01%.	The proposed framework was superior to previously tested methods. MRFO has the potential to be applied to other types of images.
[49]	BreakHis invasive ductal carcinoma (IDC) dataset		CNN with logistic regression (LR), random forest (RF), k-nearest neighbor (K-NN), support vector machine (SVM), linear SVM, Gaussian Naïve Bayesian (GNB), and decision tree (DT) processes. Hybrid DL method: CNN + classifier.	Invasive ductal carcinoma: Range accuracy: 80–86%; range precision: 92–94%; range recall: 91–96%; range F1-score: 94–96%. BreakHis: range accuracy: 91–94%; range precision: 91–95%; range recall: 93–96%; F1-score: 95–98%.	Improvements in accuracy, precision, recall, and F1 score were shown. Hyperparameter tuning, pre-processing, and image augmentation may be used to achieve better classification performance.
[1]	BreakHis	Binary and multi-class	The novel model fusion framework utilizes online mutual knowledge transfer (MF-OMKT) to classify histopathological breast cancer images. Imitates mutual communication and learning. Hybrid DL method: CNN + MF.	Accuracy range: Binary [99.27%, 99.84%]; Multi-class [96.14%, 97.53%].	The proposed framework demonstrated good accuracy. The authors suggested evaluating a fusion strategy with other cancerous image data.
[50]	BreakHis histopathological dataset—ICIAR 2018		DenseNet architecture and DenseNet architecture with image level mean. Hybrid DL method: CNN + NORM.	BreakHis: Binary DenseNet accuracy: 96.55%. Multi-class DenseNet accuracy: 91.82%. Binary DenseNet with image level mean accuracy: 91.72%. Multi-class with image level mean DenseNet accuracy: 96.72%. Competitive accuracies of 93.25% and 92.3% at the patient and image levels, respectively.	The proposed framework outperformed previously assessed methods. The normalization method seemed to influence improvements in results.
[51]	Cancer images	Not available	Wavelet transform (WT) process is applied to noisy images; VggNet-16 model Hybrid DL method: CNN + preprocessing.	Best accuracy for the dataset with Gaussian noise of a 0.3 intensity: 86.9%.	Accuracy was low. WT was not found to be suitable for image data, though it may be applicable to other types of data.

## Data Availability

Not applicable.
